# A training pipeline of an arrhythmia classifier for atrial fibrillation detection using Photoplethysmography signal

**DOI:** 10.3389/fphys.2023.1084837

**Published:** 2023-01-19

**Authors:** Sota Kudo, Zheng Chen, Xue Zhou, Leighton T. Izu, Ye Chen-Izu, Xin Zhu, Toshiyo Tamura, Shigehiko Kanaya, Ming Huang

**Affiliations:** ^1^ Computational Systems Biology Lab, Graduate School of Science and Technology, Nara Institute of Science and Technology, Ikoma, Japan; ^2^ ISIR, Osaka University, Osaka, Japan; ^3^ Department of Pharmacology, University of California, Davis, Davis, CA, United States; ^4^ Department of Biomedical Engineering, University of California, Davis, Davis, CA, United States; ^5^ Biomedical Information Engineering Lab, The University of Aizu, Aizu-Wakamatsu, Japan; ^6^ Future Robotics Organization, Waseda University, Tokyo, Japan

**Keywords:** atrial fibrillation, ectopic beats, normal sinus rhythm, deep learning, artificial neural network, model generalizability

## Abstract

Photoplethysmography (PPG) signal is potentially suitable in atrial fibrillation (AF) detection for its convenience in use and similarity in physiological origin to electrocardiogram (ECG). There are a few preceding studies that have shown the possibility of using the peak-to-peak interval of the PPG signal (PPIp) in AF detection. However, as a generalized model, the accuracy of an AF detector should be pursued on the one hand; on the other hand, its generalizability should be paid attention to in view of the individual differences in PPG manifestation of even the same arrhythmia and the existence of sub-types. Moreover, a binary classifier for atrial fibrillation and normal sinus rhythm is not convincing enough for the similarity between AF and ectopic beats. In this study, we project the atrial fibrillation detection as a multiple-class classification and try to propose a training pipeline that is advantageous both to the accuracy and generalizability of the classifier by designing and determining the configurable options of the pipeline, in terms of input format, deep learning model (with hyperparameter optimization), and scheme of transfer learning. With a rigorous comparison of the possible combinations of the configurable components in the pipeline, we confirmed that first-order difference of heartbeat sequence as the input format, a 2-layer *CNN*–1-layer *Transformer* hybrid^R^ model as the learning model and the whole model fine-tuning as the implementing scheme of transfer learning is the best combination for the pipeline (F1 value: 0.80, overall accuracy: 0.87)^R^.

## 1 Introduction

Although the symptoms of atrial fibrillation (AF) have clinical definitions and criteria, they can go unnoticed or undiagnosed due to their subtle symptoms. AF has become more prevalent in the past decade with a 3% prevalence in the adult population (Chugh et al., 2014). Since the resistance to the restoration and maintenance of sinus rhythm becomes higher as AF progresses from paroxysmal to long-standing persistence, early diagnosis and intervention are paramount (Heijman et al., 2018). In practice, AF can be accurately diagnosed with electrocardiogram (ECG) waveforms based on the invisible *p-*wave and baseline wandering (Hindricks et al., 2021). The procedure has been extended to personal care using single-lead ECG (Clifford et al., 2017).

Other than the morphological-based approach, it has been shown that the heart rate variability (HRV) extracted from the well-known R–R interval of ECG can be used in arrhythmia identification (Christini et al., 2001;Chen et al., 2021a). With an origin similar to ECG, the plethysmograph reflects the pulsation in arterial vessel/capillary. Given its physiological relation with the ECG signal, the pulse rate variability extracted from a plethysmograph is regarded as a possible surrogate of HRV in arrhythmia detection. The past few years have seen a few research works dedicated to arrhythmia detection based on the Photoplethysmography (PPG) signal (Bashar et al., 2019;Ramesh et al., 2021). For example, Bashar et al. extracted clear PPG episodes, from which the root mean square of successive differences (RMSSDs) and sample entropy were then extracted and used for classifying AF, ectopic arrhythmias, and normal sinus rhythm. Their method achieved 97% accuracy in AF vs. non-AF classification (Bashar et al., 2019). However, the individual difference in machine learning application to biomedical engineering is further magnified by the scarcity of PPG data. Therefore, while considering the natural relation between ECG and PPG signals, transferring the detection models built on the ECG signal to a new one built on the PPG signal seems plausible and indispensable for the time being.

In this regard, Ramesh et al. have tried to modify the ECG-trained model to a PPG-trained one by the transfer-learning scheme (Ramesh et al., 2021). Admittedly, the PPG signal is more vulnerable to individual differences and external influences. Skin color and blood perfusion influence the signal-to-noise ratio. Meanwhile, subtle movement of the measuring site can also distort the morphology of the PPG signal significantly. To this end, a transfer scheme that transfers the model built on a peak-to-peak interval (PPI) of ECG (PPIe) to a new model built on the PPI of PPG (PPIp) seems more feasible and robust than the transfer scheme based on waveform morphology.

Based on the aforementioned presumption and prerequisite, in this research, domain knowledge is integrated into the training pipeline to boost the performance of an arrhythmia detection model with a PPG signal and to strengthen its generalizability. Specifically, in view of the small amount of the PPG signal available, a low-dimensional input extracted from the PPIp and a lightweight deep learning model are necessary for mitigating the overfitting. Moreover, the QRS complex of arrhythmias may not necessarily be seen in the PPG signal even in a clear signal. As a result, the relevant features, engineered ones or data-driven ones, of PPIp are somewhat different from those of the PPIe. Therefore, the classification model and the transfer scheme need to be carefully designed and experimentally validated. This study, to the best of the authors’ knowledge, is the first that focuses on improving the performance and generalizability of the arrhythmia detection model based on the PPG signal by optimizing a training pipeline that is configurable in an input format, learning models, and transfer scheme. With standard pre-processing and a wide spectrum of deep learning models from basic to sophisticated ones, this study could provide a reliable training framework for robust AF detection with a PPG signal. We summarize the main contributions of this paper as the following:

• Proposal of a configurable training pipeline: we propose the pipeline by integrating the domain knowledge in physiology and machine learning;
• Construction of a lightweight *CNN–Transformer* hybrid model: we proposed a hybrid model that has not yet been tried to facilitate the model learning from both the localized segment and global context;
• Comprehensive comparison for the configurable components in the pipeline: a comparison of the 54 combinations in regard to the configurable options has been drawn to affirm the best combination that is beneficial for model performance and generalizability.

## 2 Methods

In this section, the pre-processing of ECG and PPG signals and the configurable components of the training pipeline including the input format, the selected deep learning models, and the transfer scheme are introduced as the main content. Later, the visualization of latent features in deep learning models and training and test processes are introduced concisely.

### 2.1 Datasets

#### 2.1.1 ECG datasets

PPIe samples are collected from two ECG datasets in PhysioNet (Goldberger et al., 2000): the MIT-BIH Arrhythmia Database (MIT-DB) and the Long-Term AF Database (LtAF-DB). The MIT-DB contains the excerpts of two-channel ambulatory ECGs from 47 subjects studied by the BIH Arrhythmia Laboratory (Moody and Mark, 2001). The LtAF-DB contains 84 long-term ECG recordings of subjects with paroxysmal or sustained AF, and the duration of the records is typically 24–25 h (Petrutiu et al., 2007).

From these two databases, normal sinus rhythm (NSR) samples of 30 s are extracted from episodes with normal annotations; AF samples are extracted from excerpts of AF rhythms. As frequent ectopic contractions would be recognized as AF due to the similarity in feature space (Chong et al., 2015), ectopic (PVC/PAC) samples are also extracted from episodes with normal and PVC/PAC annotations.

#### 2.1.2 PPG datasets

As for the PPG signal with arrhythmia annotation, the UMMC Simband Dataset (UMMC-DB) is used. Specifically, the UMMC-DB contains simultaneous ECG and PPG records of 41 patients with cardiac arrhythmia (AF and PAC/PVC). The records are segmented into 30-s annotated samples. Table 1 shows the statistics of the subjects and samples extracted from each dataset.

**TABLE 1:** Statistics of subject numbers and sample numbers of the datasets used in this research.

Dataset	# Subject	# NSR	# AF	# Ectopic
LtAF	84	52407	50890	41263
MIT	47	798	68	1097
UMMC	37	192	54	42

### 2.2 Pipeline

#### 2.2.1 Pre-processing of datasets

Based on the presumption that model transfer learning from ECG to PPG boosts the performance of AF detection using the PPG signal, PPIe and PPIp are used as the source information of classification models. R peaks are picked out with the modified version of the Pan–Tompkins algorithm, used in a preceding study (Datta et al., 2017). For the PPG dataset, raw PPG samples were filtered with a sixth-order Butterworth filter with 0.5 Hz and 5 Hz cutoffs. Later, the peak detection method designed by Elgendi (2013) was used to generate PPIp of each sample. The PPIe and PPIp sequences were then converted to integral heart rate (HR) sequences.

#### 2.2.2 Model input

Differences in HR sequence between heartbeats are used as the input of the selected models that will be introduced later. Physiologically speaking, the differences in HR have been used to characterize pathological conditions of the heart for being able to access the non-linear dynamics of the beat-to-beat interval, and its effectiveness in characterizing arrhythmia has been validated by preceding research (Zhang et al., 2015;Park et al., 2009). The reason for choosing the difference in HR as the input also lies in that temporal information that may be important in classification is still available in this form, while the sample-wise statistics wipes out all temporal information. There are three types of HR differences deemed to be suitable here.

• *input1*: first-order differences, which consist of the difference in HR between the current heartbeat and its adjacent heartbeats (two-dimensional);
• *input2*: first-order differences and the current HR (three-dimensional);
• *input3*: first-order differences and second-order differences, which use the HR of the same three consecutive heartbeats as used in the first-order difference (three-dimensional).

#### 2.2.3 Models

In addition to the recurrent neural network (*RNN*), which is tailored for sequential learning (Sak et al., 2014), the convolutional neural network (*CNN*) is also appropriate in PPI-based feature extraction because a localized pattern in heartbeat sequence is important in arrhythmia recognition (Olier et al., 2021). Moreover, the attention-based model, e.g., the *Transformer* (Vaswani et al., 2017), has reached state-of-the-art (SOTA) performance in a variety of fields of sequential data (Zhao et al., 2020;Chen et al., 2021b). Therefore, *Transformer* and the hybrid variant were also adopted. In this paper, we recap the theoretical part of these three layers as follows:

***LSTM* layer**: *LSTM* has an input *x*(*t*) which can be the output of a *CNN* or the input sequence directly. *h* (*t*−1) and *c* (*t*−1) are the inputs from the previous time step. *o*(*t*) is the output of the *LSTM* for this time step. The *LSTM* also generates the *c*(*t*) and *h*(*t*) for the consumption of the next time step.

$$f_{t}=\sigma_{g}\left(W_{f}\times x_{t}+U_{f}\times h_{t-1}+b_{f}\right),$$ (1)

$$i_{t}=\sigma_{g}\left(W_{i}\times x_{t}+U_{i}\times h_{t-1}+b_{i}\right),$$ (2)

$$o_{t}=\sigma_{g}\left(W_{o}\times x_{t}+U_{o}\times h_{t-1}+b_{o}\right),$$ (3)

$$c_{t}^{\prime }=\sigma_{c}\left(W_{c}\times x_{t}+U_{c}\times h_{t-1}+b_{c}\right),$$ (4)

$$c_{t}=f_{t}\cdot ct-1+i_{t}\cdot c_{t}^{\prime },$$ (5)

$$h_{t}=o_{t}\cdot \sigma \left(c_{t}\right),c_{t},$$ (6)

where *f*_*t*_, *i*_*t*_, *o*_*t*_, *c*_*t*_, and *h*_*t*_ are the forget gate, input gate, output gate, cell state, and hidden state, respectively.

***CNN* layer**: The *CNN* layer extracts the localized information from the 1-day/2-day data by implementing the 1-day/2-day convolution throughout the sequence.

$$s\left[t\right]=\left(x\;*\;w_{\mathrm{cov}}\right)\left[t\right]=\overset{a=\infty }{\underset{a=-\infty }{\sum }}x\left[a\right]w_{\mathrm{cov}}\left[a+t\right],$$ (7)

where *s* [*t*] denotes the feature map that is generated by the kernel mapping *w*_cov_ of the *CNN* layer.

***Transformer***: Recently, the *Transformer*, which is a full attention-based model, reaches SOTA in a variety of computational tasks. The attention mechanism pays greater attention to parallelly seeking the salient factors, and it is competent for sequence modeling of dependencies without considering the information transfer of time step. Here, the *Transformer* is introduced as the backbone of our framework that aims to summarize the feature relevance for a specific problem. For each sample, a preparation that is to append an extra class token *S*_*Cls*_ to the front of each PPI sample is required. Because the *Transformer* model practically does not derive a new latent feature for the downstream task, this *S*_*Cls*_ is generated as the latent variable. This token absorbs the global context of features with the attention calculation for the input. Afterward, it is used to generate downstream decision rules for heartbeat rhythm recognition.

The input (*X*) of the *Transformer* can be formulated as follows:

$$X=Concat\left(S_{\mathrm{Cls}},E\cdot S_{\mathrm{seq}}\right)+f_{P}\left(E_{\mathrm{pos}}\right),$$ (8)

where *E* is a patch-wise linear projection that expands the feature space of input to a higher dimension. Before input to the *Transformer* model, a parameterizing operation of positioning (*E*_*pos*_) of the input features is needed for all elements of the sequence.

*Attention mechanism:* The attention mechanism associates the individual and maps the relevance to the ground truth *y* with three components: the query (*Q*), key (*K*), and value (*V*) matrices, which are the matrix of linear projections produced by the input *X*. The matrix *Q* represents a query that comprises a query sequence with basic units. Moreover, the output of *K* ⋅ *Q* produces relevance among all elements of the sequence, and the function Softmax is used to calculate weights of this relevance. The resultant relevance values are further used to calculate *V*. The aforementioned process can be summarized as follows:

$$Score_{\mathrm{Attention}}=\text{Softmax}\left(\frac{QK^{T}}{\sqrt{d}}\right)\cdot V,$$ (9)

where layer $\sqrt{d}$ is equal to a normalization function that is applied to each *Q*–*K* calculation step.

*Multi-head attention:* Similar to the way that a *CNN* increases the number of filters to enrich the expressiveness of the feature space, the attention mechanism can be extended to multi-head attention (*Z*_*Mhead*_) to prevent losing the manifold expression of the features. At the beginning of each building block, *h* (the number of heads) sets of *Q* and *K* are generated and mapped by the linear projection. Then, the self-attention implements *h* times in parallel to calculate relevance representations, where each operation is called a “head.” Eventually, a linear layer projects their concatenated outputs and summarizes the attention result. The multi-head attention is defined as follows:

$$Z_{\mathrm{Mhead}}\left(Q,K,V\right)=\text{Concatenate}\left(head_{1},head_{2},\ldots ,head_{h}\right)W^{o},$$ (10)

where $W^{o}\in R^{h\cdot D\times \left(150+1\right)}$ is a weight matrix. It is used for head-wise attention, while a linear projection is applied after the output of the multi-head attention for each round. Since this work aims to build a correspondence between the input PPG sample to AF and other cardiac rhythms, the final output of the *Transformer* is the classified possibilities of the AF, PVC, and NSR.

$$y^{\prime }=\text{Softmax}\left(\beta \left(\cdot \right)\left(S_{\mathrm{Cls}}^{\prime }\right)\right),$$ (11)

where *y*′ ∈ {AF, PVC, NSR} and $S_{\mathrm{Cls}}^{\prime }$ are also normalized before the final classification layer, where *β*(⋅) denotes a LayerNorm operator.

Based on the aforementioned basic layers, six deep learning models, which are deemed appropriate, have been constructed. They are as follows:

• Long–short-term memory (*LSTM*) model (m1);
• *CNN* model (m2) and its variants: *CNN*-based *inception* model (m3) and *CNN*–*LSTM* hybrid model (m4);
• *Transformer* model (m5) and its variant (*CNN*–*Transformer*) hybrid model (m6).

**FIGURE 1:**
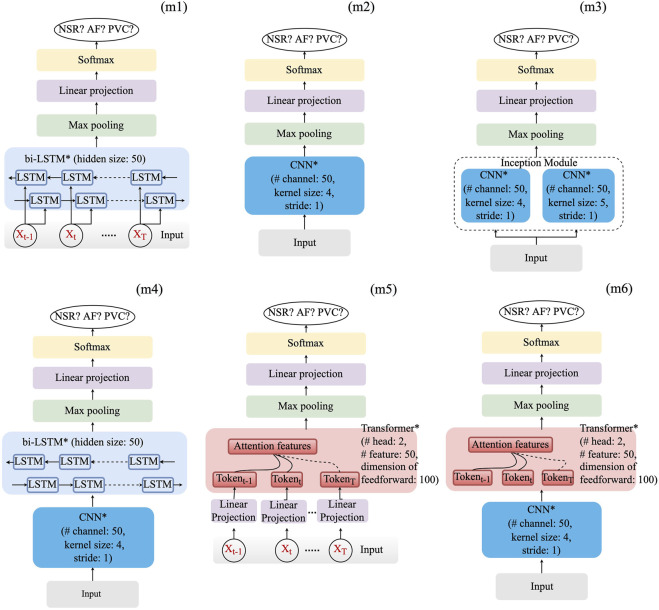
Structure of the six selected model frameworks. The number of layers whose name is followed by an asterisk is configurable. The values, e.g., # head of the *Transformer*, which are shown in layers, are fixed.

As shown in Figure 1, optimization of the model structure, in terms of hyperparameters such as layer number and learning rate of the optimizer, was conducted. Hyperparameters, for which grid searching was conducted, are summarized in Table 2.

**TABLE 2:** Hyperparameters of grid search. The learning rate and the weight decay are used in the optimizer (Adam) initialization, and the drop rate (last hidden layer only) is used in the model regularization. The *dense* layer is used to rearrange and project the latent features to generalize the decision rule, and the value inside the parenthesis shows the dimension of the layer.

Hyperparameter	PPIe	PPIp
# *CNN* layer	[1:1:5]	[1:1:5]
# *LSTM* layer	[1:1:5]	[1:1:5]
# *Transformer* layer	[1:1:5]	[1:1:5]
# *Dense* layer	1 (50)	1 (50)
Epoch	5	50
Learning rate	0.001	0.001
Weight decay	0.0	0.0
Dropout rate	0.0	0.2

#### 2.2.4 Transfer learning schemes

Generally, as the layers of a deep learning model goes deeper, its neurons become more specific to the problem. However, as we have pointed out in *Introduction*, the PPIe is somewhat different from the PPIp in case of arrhythmia. A suitable way to implement transfer learning should be discussed. In this study, two transfer schemes, 1) transfer learning of the last layer (TS1) and 2) transfer learning of all the deep layers (TS2) were set up. Basically, the last one or two layers of a deep learning model, which are typically *dense* layers, are used to rearrange the latent features extracted by the upstream layers (feature extraction layers) and to generate the decision rule for classification problems. The TS1 implemented a transfer learning in the last layer with the presumption that the way of feature extraction learned from the ECG signal is appropriate for the PPG signal. In contrast, TS2 presumes that the feature extraction should be further optimized. Consequently, the whole model, including the feature extraction layers and the decision rule generating layers, was further optimized for the PPG signal.

#### 2.2.5 Training and test

**FIGURE 2:**
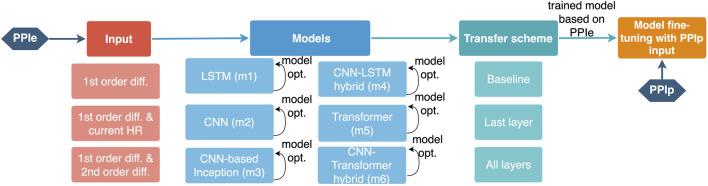
Illustration of the training pipeline for a PPIp-based arrhythmia detection model. The configurable components are shown with their options below the corresponding component.

As shown in Table 1, moderate data imbalance appears in the LtAF-DB and UMMC-DB, whereas severe imbalance appears in the MIT-DB. Therefore, the LtAF-DB and MIT-DB were used as the training and validation datasets, respectively, in the training process with PPIe data, during which weighted random sampling (Paszke et al., 2019) is used to mitigate the influence of data imbalance in the LtAF-DB. To strengthen the generalizability of a model, it should be exposed to the influence of individual differences. Therefore, leave-one-subject-out (loso) cross-validation is taken in the fine-tuning process with PPIp data. The overall process is shown in Figure 2.

The F1 score, which is the harmonic mean of recall and precision, considers the trade-off between the false-positive and false-negative. Therefore, it is suitable for evaluating the model performance with imbalanced data. In this research, the F1 score is used as the primary metric of model evaluation, along with which other metrics in the confusion matrix will also be used to compare the best model in each transfer scheme (TS1, TS2, and baseline (no-transfer)).

#### 2.2.6 Visualization

A clear separation between classes in the feature space will benefit the generalizability of a model by mitigating the influence of overfitting of the decision rule (Amjad and Geiger, 2020). For the best model, the output of the neurons in the middle (for example, the last *CNN* layer in the *CNN*–*Transformer* hybrid model) and last layers were extracted from both the pre-trained and fine-tuned models. With t-SNE, we implemented dimension reduction of the output to confirm the similarity of sample distribution and concentration in feature spaces of PPIe and PPIp. It could support our presumption that transfer learning benefits the performance and generalizability of the AF detection model based on the PPIp.

## 3 Results

As introduced in the previous section, the model trained with the LtAF-DB is validated with the MIT-DB; the pre-trained model was then fine-tuned with the UMMC-DB and tested by loso cross-validation. The results of validation with the loso test are shown side-by-side in Table 3. The three-column blocks for each transfer scheme are summarized alongside the pre-train column block. The three columns in each block correspond to the best results with *input1*, *input2*, and *input3*, respectively. The three items of each entry in the table from top to bottom refer to the F1 value, accuracy, and hyperparameters (layer number(s)), respectively. Intriguingly, the best hyperparameters vary between pre-trained and fine-tuning models. For example, the model (m6) with PPIp requires two *CNN* layers, while that with PPIe requires just one. As the bold values in each block show the best model input combination, TS2 attains the best performance (F1 = 0.80) with the m4 and m6 models. With the same F1 values and similar overall accuracy values, the m6 model is chosen over the m4 model because the m6 model has a higher precision value (0.79) than that of the m4 model (0.75), while the recall values are the same as 0.90. This result also implies the ablation of second-order differences does not significantly change the accuracy. Therefore, we choose *input1* as the best input.

**TABLE 3:** Best models with different inputs. The three items of each entry in the table from top to bottom refer to the F1 value, accuracy, and hyperparameters (layer number(s)), respectively. Notably, the best model with PPIe and that with PPIp may differ in terms of the number of layers. For the hybrid type, the x–y denotes the number of the former and latter main layers, respectively. m1, *LSTM* model; m2, *CNN* model; m3, *CNN*-based *inception* model; m4, *CNN–LSTM* hybrid model; m5, *Transformer* model; and m6, *CNN–Transformer* hybrid model.

Model	PPIe						PPIp					
	Pre-train			Baseline			TS1			TS2		
m1	0.78	0.77	0.77	0.63	0.55	0.64	0.69	0.72	0.67	0.78	0.72	0.76
	0.87	0.85	0.87	0.73	0.69	0.74	0.80	0.80	0.78	0.88	0.85	0.86
	3	3	2	1	1	5	3	2	2	5	4	5
m2	0.80	0.81	0.78	0.69	0.65	0.66	0.73	0.68	0.73	0.78	0.71	0.79
	0.89	0.88	0.88	0.80	0.77	0.78	0.82	0.80	0.83	0.86	0.84	0.87
	2	3	2	3	4	3	3	5	5	3	5	3
m3	0.79	0.77	**0.83**	0.68	0.68	0.68	0.72	0.72	0.76	0.76	0.67	0.74
	0.88	0.86	**0.90**	0.80	0.79	0.78	0.81	0.81	0.82	0.86	0.78	0.83
	5	5	**2**	5	3	2	3	5	4	3	2	2
m4	0.81	0.81	0.79	**0.70**	**0.70**	**0.70**	0.74	0.71	0.74	0.79	0.75	**0.80**
	0.89	0.87	0.88	**0.77**	**0.80**	**0.79**	0.81	0.77	0.82	0.87	0.85	**0.88**
	2–1	1–5	4–1	**1–1**	**2–5**	**2–1**	3–1	3–2	5–5	3–3	3–3	**2–2**
m5	0.81	0.74	0.80	0.61	0.56	0.58	0.68	0.71	0.66	0.78	0.66	0.68
	0.88	0.85	0.88	0.75	0.64	0.73	0.78	0.77	0.76	0.85	0.83	0.80
	3	1	2	4	1	2	3	3	5	5	3	2
m6	0.82	0.81	0.80	0.67	0.66	**0.70**	0.75	**0.77**	0.72	**0.80**	0.75	0.78
	0.90	0.89	0.88	0.78	0.78	**0.82**	0.84	**0.83**	0.80	**0.87**	0.85	0.87
	1–1	3–2	4–1	3–1	5–1	**2–3**	4–4	**2–1**	4–2	**2–1**	2–1	3–1

The performance of models with PPIe data is generally better than the ones using PPIp data even when fine-tuning was implemented. It supports our presumption that the clear differences among the targeted arrhythmia and NSR rhythm in PPIe patterns could become faint in PPIp due to the uncertainty in arrhythmia manifestation.

**FIGURE 3:**
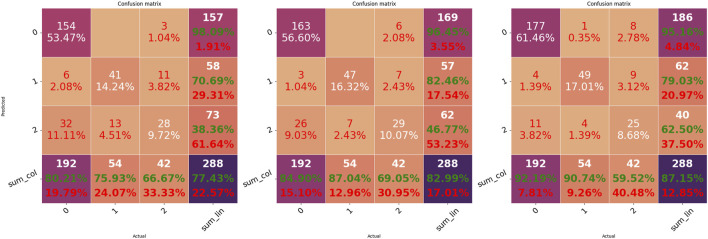
Confusion matrices of the three transfer schemes. Matrices from left to right indicate the results of the best model corresponding to baseline (m4)^R^, TS1 (m6)^R^, and TS2 (m6), respectively.

Intriguingly, there is no evident performance improvement after pre-training the model using the TS1 scheme (TS1 vs. baseline). However, the TS2 scheme obtained significantly better results for almost all models than the TS1 scheme and baseline. For each scheme, the performance of the best model is further displayed by its confusion matrix, as shown in Figure 3. Along with the accuracy, it can be seen that the TS2 scheme achieves the best performance with an accuracy of 0.87. As anticipated, ectopic arrhythmia behaves as a confounding factor in AF detection. For example, the inconsistency of beat detection in ECG and PPG happens in situations such as trigeminy. Consequently, the beat that cannot be picked out in PPG blurs the distinction between the AF and ectopic type.

**FIGURE 4:**
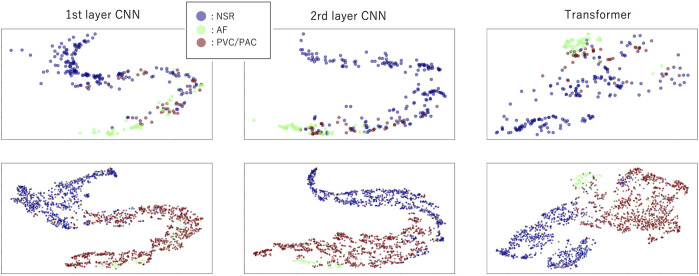
Distribution of the latent features in model (m6) layers after dimension reduction with t-SNE. The upper row indicates latent features in layers using PPIp, and the lower row shows the latent features using PPIe. The first two columns are the features of the first and the last *CNN* layers, respectively, while the third column is the features of the last *transformer* layer.

Figure 4 shows the visualization of the distribution of latent features in the *CNN–Transformer hybrid* model before fine-tuning with PPIp data. Without the fine-tuning step, the PPIp samples are transformed in exactly the same way as the PPIe samples, by which the PPIp samples of the three heartbeat types can also be organized into dense regions. For both the cases of PPIe (lower row) and PPIp (upper row), as the layer goes deeper, the sample distribution of each class becomes more separated generally. This observation suggests the availability of the pre-trained model with PPIe data in constructing the model with PPIp samples. However, although being mitigated, the problem that a portion of ectopic samples overlaps with AF samples still exists.

**FIGURE 5:**
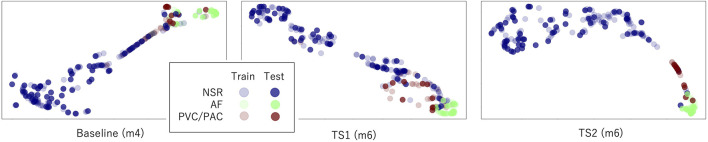
Distribution of the latent features in the last layer of the best model of each transfer scheme. *Input1* is used here.

The latent features of the best model of each transfer scheme are further visualized in Figure 5 using the data from nine subjects in the UMMC-DB. In contrast with the mix-up of the AF and ectopic samples in baseline and TS1 situations, the AF samples are generally well separated from samples of the other types in TS2. In addition, the inconsistency in the distribution of the training and testing samples was confirmed. For example, for TS2, a couple of ectopic samples of the PPIp test^R^ set are found close to the AF samples of the training^R^ set. This observation is prevalent for physiological signals and may be caused by individual differences or similarities between the ectopic and AF episodes.

## 4 Discussion

### 4.1 Physiological perspective

Features from other domains, such as the entropy domain, have been shown to be sensitive to different heart rhythms (Christini et al., 2001). Chen et al. have shown that a variant of the multiscale entropy provides informative features for heartbeat rhythm classification (Chen et al., 2021a). However, after the preliminary validation stage using the entropy features for this problem, we confirmed that due to the short length of the PPG sequence (30 s), very limited features, only the first two or three scales, can be computed; they are not sufficient for the current problem.

The first-order difference of the HR sequence attains the best performance in our comparison. It is in line with the preceding studies that show good classification results using the same input (Ramesh et al., 2021). However, we have also confirmed the uncertainty in using this input, e.g., the Poincare plot, to discriminate the rhythm in both PPIe and PPIp. Specifically, the clear difference between AF and ectopic beats in the pattern of the Poincare plot (Han et al., 2020) does not necessarily exist. The uncertainty can also be confirmed from the results of the models with a shallow network (Li et al., 2020), e.g., 1-layer *LSTM* and 1-layer *CNN* trained without transfer learning. These simple models are sufficient to conclude a rule of thumb for arrhythmia classification. However, these models did not attain results comparable with those of the other constructed models. Given the aforementioned situation, this study is conducted to look for a more capable and robust pipeline for arrhythmia detection using a PPG signal.

As we can see from Figure 4, there is a clear separation between NSR and AF in both PPIe and PPIp. However, when the ectopic ones are mixed in, the separation between both NSR and AF is vague. This phenomenon is not only confined to the input format in our study, other research with information entropy as its input has also reported a similar observation (Pereira et al., 2020; Chen et al., 2021a). The algorithm used in this study for ECG peak detection is well-established, and the peak detection has been manually checked by a technician. On the other hand, although the algorithm of peak detection of PPG still needs improvement, the systolic PPG peaks were picked out by a peak detection method (Elgendi, 2013) that is broadly accepted for the PPG signal; thereafter, they got a manual check with reference to the paired ECG signal. Piecing together all the aforementioned information, it can be concluded that erroneous peak detection is not the main reason for the overlapping of sample distribution in the latent feature space, which we further discuss.

### 4.2 Machine learning perspective

Understandably, uncertainty is even stronger in the biomedical engineering domain and it exists in almost all aspects, from the anatomical/physiological differences to the differences in devices, the differences in data pre-processing, *etc.* Specific to arrhythmia detection using a PPG signal, the automated peak detection algorithm also introduces uncertainty *via* the erroneously detected peaks. Currently, the peak detection for the PPG signal is still being improved; therefore, instead of removing the erroneous peaks, they are retained in the training/testing dataset. Theoretically, they influence the training process from feature extraction to decision rule generation.

Since the model is trained substantially on the PPIe dataset, it can be seen from Figure 4 that the distribution of PPIp samples feature space is generally similar to the distribution in PPIe feature space. Moreover, the gradual transformation of the features along the deep layers drives the samples of the same class to distribute closer together. This observation is a visual confirmation of our presumption that the transfer learning from PPIe to PPIp is beneficial for the general performance of the classification model based on the PPG signal. Other than the PPI sequence, preceding research has also tried using PPG morphological information in arrhythmia detection (Väliaho et al., 2021). However, in this way, a domain adaption model (Chen et al., 2022), for which a great amount of ECG–PPG paired samples are demanded, is needed in order to take in the information from PPIe.

In this study, there are three major layers used in the selected models, the first two of which are the *LSTM* layer and *CNN* layer. The *LSTM* layer itself can learn to combine the information of each element in the sequence; therefore, it was widely used in sequence learning before the advent of the *Transformer*. The *CNN* layer is theoretically close to the convolution in signal processing and is designed to extract the feature in a segment. Given that the short segment of beats such as trigeminy shows a specific pattern, the *CNN* layer is used as the first layer(s) of the hybrid models. The third kind of layer, the *attention* layer in the *Transformer* model tries to find the global element-wise relation in parallel, in contradiction to the sequential combination of information taken by *LSTM*. Therefore, while the important information embedded in elements being far apart from each other may become faint in *LSTM*, it can be captured by *attention*. In considering that the *CNN–Transformer* hybrid model gets the best results with the TS2 scheme, it implies that the PPIe samples provide additional information in using the overall context of PPI sequence in classification.

Again, this study is not going to draw a direct comparison of the model performance with other papers because the differences in peak detection, sample inclusion criteria, *etc.,* could have a considerable impact on the results. As discussed earlier, we specify the problem to strengthen the generalizability of the arrhythmia classifier using the PPG signal by answering the following questions: 1) Is the transfer learning from a model that uses the ECG signal to another model that uses the PPG signal necessary? And 2) how to implement transfer learning? To this end, a rigorous comparison of the possible combinations of configurable components in the training pipeline was conducted (Figure 2).

According to the universality of the neural networks, if the distributions of the training and test sets are highly similar, a shallow network with one or two *dense* layers can sufficiently approximate the decision function to summarize a perfect decision rule (Bishop, 2006). However, as can be seen in the performance of models, no model can output a very accurate result even when the sample distributions of each type are separated. The sample distribution shown in Figure 5 may explain this disparity. For example, in the m6 model (right sub-figure), some test samples of the ectopic type disperse in the region occupied majorly by AF samples (lower right corner). These ectopic samples will be understandably recognized as AF samples. A similar situation appears in the ectopic region, where NSR samples show up. Therefore, the inconsistency between the sample distributions of the training and test sets could be the main reason for the imperfect performance of all models.

The observation of disparity between TS1 and TS2 is in accordance with the observation of PPIe and PPIp in arrhythmias. Therefore, PPIe and PPIp may need different filters for feature extraction, i.e., different *CNN* layers at the very beginning. On the other hand, using the parameters in the pre-trained model, each model can find a better local optimum than the baseline situation. This point is also reflected in Table 3, where TS2 is better than the baseline situation in all models. As discussed earlier, the pre-training with PPIe provides a better initialization on one hand. On the other hand, it also acts as a restraint that keeps the model from overfitting for external data. Therefore, the transfer scheme seems necessary in training an arrhythmia classifier based on the PPG signal even when the PPG data got fast accumulated.

### 4.3 Conclusion

In this study, an efficient training pipeline is designed and developed for training a robust arrhythmic classifier for AF detection using the PPG signal. The most efficient pipeline is drawn by determining the configurable components which are the input format, the deep learning model, and the transfer scheme. The first-order difference of heartbeat sequence, a 2-layer *CNN*–1-layer *Transformer* hybrid^R^ model, and the whole-model fine-tuning turn out to be the best combination for the pipeline with a 0.80 F1 value and a 0.87 accuracy^R^. The pipeline is determined by incorporating the standard pre-processing in the ECG and PPG signals, domain knowledge in the application of physiological signals, and advanced deep learning models for feature learning and decision rule drawing. Although the performance of the classifier may vary with other peak detection methods for a specific dataset, the proposed pipeline is useful in training an accurate and robust arrhythmia classifier.

## Data Availability

Publicly available datasets were analyzed in this study. These data can be found here: https://physionet.org/content/mitdb/1.0.0/https://physionet.org/content/ltafdb/1.0.0/https://www.synapse.org/#!Synapse:syn23565056/wiki/608635.
